# Plant management and biodiversity conservation in Náhuatl homegardens of the Tehuacán Valley, Mexico

**DOI:** 10.1186/1746-4269-9-74

**Published:** 2013-11-06

**Authors:** Carolina Larios, Alejandro Casas, Mariana Vallejo, Ana Isabel Moreno-Calles, José Blancas

**Affiliations:** 1 Centro de Investigaciones en Ecosistemas, UNAM, Antigua Carretera a Pátzcuaro 8711, Col. San José de la Huerta Morelia, Michoacán 58190, México; 2 Escuela Nacional de Estudios Superiores, UNAM, Antigua Carretera a Pátzcuaro 8711, Col. San José de la Huerta Morelia, Michoacán 58190, México

**Keywords:** Biodiversity conservation, Domestication, Homegardens, Náhuatl people, Plant management, Sustainable use, Tehuacán Valley

## Abstract

**Background:**

The Tehuacán Valley is one of the areas of Mesoamerica with the oldest history of plant management. Homegardens are among the most ancient management systems that currently provide economic benefits to people and are reservoirs of native biodiversity. Previous studies estimated that 30% of the plant richness of homegardens of the region are native plant species from wild populations. We studied in Náhuatl communities the proportion of native plant species maintained in homegardens, hypothesizing to find a proportion similar to that estimated at regional level, mainly plant resources maintained for edible, medicinal and ornamental purposes.

**Methods:**

We analysed the composition of plant species of homegardens and their similarity with surrounding Cloud Forest (CF), Tropical Rainforest (TRF), Tropical Dry forest (TDF), and Thorn-Scrub Forest (TSF). We determined density, frequency and biomass of plant species composing homegardens and forests through vegetation sampling of a total of 30 homegardens and nine plots of forests, and documented ethnobotanical information on use, management, and economic benefits from plants maintained in homegardens.

**Results:**

A total of 281 plant species was recorded with 12 use categories, 115 ornamental, 92 edible, and 50 medicinal plant species. We recorded 49.8 ± 23.2 (average ± S.D.) woody plant species (shrubs and trees) per homegarden. In total, 34% species are native to the Tehuacán Valley and nearly 16% are components of the surrounding forests. A total of 176 species were cultivated through seeds, vegetative propagules or transplanted entire individual plants, 71 tolerated, and 23 enhanced. The highest species richness and diversity were recorded in homegardens from the CF zone (199 species), followed by those from the TRF (157) and those from the TDF (141) zones.

**Conclusion:**

Homegardens provide a high diversity of resources for subsistence of local households and significantly contribute to conservation of native biodiversity. The highest diversity was recorded in homegardens where the neighbouring forests had the least diversity, suggesting that management of homegardens aims at compensating scarcity of naturally available plant resources. Cultivated species were markedly more abundant than plants under other management forms. Diversity harboured and management techniques make homegardens keystones in strategies for regional biodiversity conservation.

## Background

Homegardens are important agroforestry systems developed by numerous human cultures worldwide [1]. Characteristically located attached to peoples’ houses, these systems are commonly formed by a variety of plant and animal species either wild and domesticated, whose composition and structure are continually transformed according to plans designed by humans that manage them [1-7]. These processes illustrate mechanisms of domestication operating at ecosystems and landscape levels [8,9]. Homegardens commonly are reservoirs of agrobiodiversity but also they may maintain native natural biodiversity [6,8], including genetic diversity of species occurring wild in forests [10-12]. This is possible since local people that manage the system frequently carry to homegardens plants from the wild [13-18], which favours gene flow from wild and cultivated components [8,9,12,17-19] and ecological processes similar to those occurring in the surrounding forests. All these aspects in theory confer to homegardens a high resilience capacity [1].

People that manage homegardens find in them multiple goods to satisfy their social, cultural and economic needs, mainly food, medicines, ornamental and spiritual wellbeing, fodder, fuel wood, and products that generate monetary incomes [13,20-22]. Several authors have documented that these systems are also areas where domestication is experimented and agricultural practices are commonly tested there before carrying out them into parcels in fields out of the villages [6,9,12,15,17,23]. Since homegardens are spaces of resources, management techniques, and human cultural processes these systems are considered as important reservoirs of biocultural heritage [24,25].

The great variety of products provided by homegardens occurs in areas relatively small. According to Van der Wal and Bongers [26] homegardens in rural regions of Mexico may be ‘small’ (less than 1,000 m^2^), ‘intermediate sized’ (1,000 m^2^ to 2,000 m^2^), and ‘large’ (more than 2,000 m^2^), which indicates that such high diversity maintained in small areas necessarily involves strategies for optimizing usage of space and resources such as light, nutrients and water.

Several authors have questioned the capacity of traditional agricultural systems for reaching the challenges of productivity required for feeding the global society, suggesting that it is only through intensive industrialized systems that such purpose can be accomplished; however, it is real that the intensive industrialized systems have failed in numerous contexts and that their achievements have been accompanied with high environmental costs [27]. Therefore, looking for strategies for improving capacities of the traditional systems has become a new paradigm for constructing sustainable security systems of food and other goods for human life [28].

The purposes of increasing productivity has commonly promoted the simplification of traditional agroforestry systems, consequently leading to lose some of the principal attributes of sustainability [7,29]. One of the greatest challenges of the contemporary human societies is therefore how to achieve optimum productivity without losing diversity of components and functions of these systems. According to an increasing number of authors [5,27], productivity and sustainability are concealable properties of agroecosystems through agroecological principles. Documenting local management experiences, therefore, has become a primary source of empirical information for developing theory about such important principles. This is what our current study looks contributing for.

Different indigenous cultures in Mesoamerica have conserved traditional ecological knowledge and forms of natural resources management [21,24,25,30,31], which represent thousands of years of adaptation of human groups to particular surrounding environments and confer to them a high potential contribution for sustainable socio-ecological systems and biodiversity conservation [32,33]. However, traditional knowledge and techniques are currently endangered and in process of gradual disappearing throughout the World [24,25]. Promotion of modern techniques considered as having higher effectiveness and cultural prestige, migration, unemployment favouring abandonment of agricultural practices, bad governmental assistance policies, fragmentation of land tenure, among others are all factors influencing losing of traditional systems of resource management [7,29,31].

The Tehuacán Valley is one of the arid zones with the highest biodiversity of the Americas [34]. Although few studies are still available on agroforestry systems of that region, it has been documented that these systems, among them homegardens, harbour high native biological diversity [6,7,29], and could be key targets for policies of biodiversity conservation at regional level [6,18]. More studies are needed for constructing such a strategy; for instance, only one case study of homegardens [20] has been reported in the literature for the region. Natural resources managed in agroforestry systems in general and homegardens in particular could be targets for improving conditions of human life and for maintaining ecosystem services. Socio-ecological sustainability should include ecological, social and economic dimensions [35,36] and therefore, homegardens as systems complementing ecological functions and households wellbeing are important bases for designing socio-ecological sustainable ways of life [37]. An agroforestry system is more probably ecologically sustainable when allows biodiversity conservation and maintenance of water and soil, which in turn favours diversity of biotic interactions buffering changes in temperature and humidity, maintenance of nutrients cycling, efficient use of light and waste management determining wellbeing of people that manage them [37,38].

Our study focused on determining composition of homegardens in Náhuatl rural communities, evaluating the capacity of these systems for conserving native biodiversity and their role in households’ economy, but we particularly emphasized documenting the local management techniques since we value them as crucial human experience for explaining past processes and the current state of problems, as well as for designing future management strategies [17]. Previous studies in the region have identified general forms of plant management in agroforestry systems, such as tolerance, promotion, protection, and cultivation [6,9,17,23,39]. In homegardens, tolerated plants are those growing ‘spontaneously’ (not human mediated but by natural propagation means) and that people let standing deliberately since they obtain a benefit (direct use or service) or because presence of plants do not cause any damage. Plants promoted or enhanced are those tolerated, already occurring in homegardens, and that people deliberately propagate by sowing their seeds or planting their vegetative propagules or entire young plants with the purpose of increasing their availability. People also use to protect especially those tolerated plants that are particularly valuable; for instance, they may provide structures for appropriate growing, construct irrigation systems to benefit them, take actions for protecting plants against herbivores, or prune neighbouring plants to let sun light reaching the protected plants. Cultivation involves sowing or planting of plants that were not naturally in homegardens areas and that people bring to them from the wild or from other agricultural systems [6,9,23].

Studies of traditional agricultural systems in tropical regions of the world provide important information for understanding ecological processes associated to sustainable management of natural resources [40]. Agroecology, according to Gliessman [40], is the application of concepts and principles of ecology for sustainably designing and managing agroecosystems; consequently, our study looks for understanding cultural and ecological principles connecting explicitly the value of ethnobiological approaches for understanding structure and functions of homegardens at local scale in order to identify bases for designing strategies of their sustainable management at both local and regional levels.

We studied homegardens managed by the Náhuatl people of communities at Coyomeapan, and Coxcatlán Puebla, in the Tehuacán Valley, analysing their capacity to maintain native species and their possible role in policies for biodiversity conservation and wellbeing of local people. We hypothesized that since native plant species are continually introduced to homegardens by people, plant diversity harboured in these systems would be proportional to the natural diversity existing in local forests, and also similar to the proportion of native plant species found in homegardens at regional level; we also expected that native species were mainly represented by components of the neighbouring natural vegetation within the territory of a village. We documented the benefits local people obtain from managing these systems, particularly those of the native species. In this respect we supposed that the primary aims directed to manage homegardens is easing access to edible, medicinal and ornamental plants, in this order. And finally, we documented plant management involved in homegardens, expecting higher frequency of tolerance and transplanting as found in other agroforestry systems of the communities studied. Our study aimed at: (1) inventorying plant species occurring in homegardens, their nomenclature, use and traditional management, (2) determining richness, abundance and diversity of plant species composing homegardens, and their role in maintaining native plant species, and (3) evaluating harvest, consumption and incomes obtained from homegardens’ products and comparing the role of this system in people’s subsistence and culture in different ecological conditions.

## Methods

### Study area

**Figure 1:**
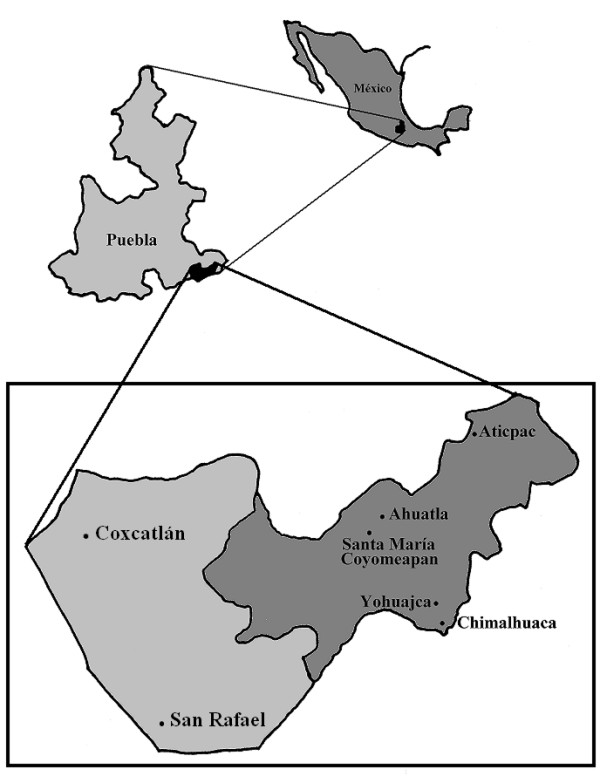
**Study area.** Communities studied in the municipalities of Coyomeapan and Coxcatlán in the state of Puebla, central México.

We studied homegardens from villages of the municipalities of Coyomeapan and Coxcatlán, located at the southeast of the state of Puebla in central Mexico (Figure 1). In the municipality of Coyomeapan we studied the communities of Coyomeapan at elevations averaging 2800 m, Ahuatla at 2400 m, Yohuajca at 2200 m, Chimalhuaca at 1840 m, and Aticpac at 1140 m. In these communities the predominant vegetation is distributed in three main environmental zones as follows: (1) Cloud Forest Zone (CFZ) in Coyomeapan and Ahuatla, (2) Tropical Rainforest Zone (TRFZ) in Aticpac, and (3) Tropical Dry Forest Zone (TDFZ) in Chimalhuaca and Yohuajca. In addition, we included in our analysis information from homegardens of a Thorn-Scrub Forest Zone (TSFZ) previously studied by Blanckaert *et al*. [20] in the village of San Rafael, in the municipality of Coxcatlán, neighbouring to Coyomeapan at 1200 m of elevation; also, we considered the information from the natural vegetation surrounding this village and studied by Vivar [41]. People of the communities studied live based on agriculture practiced in traditional agroforestry systems in fields out of the villages [7,29], as well as managing homegardens, raising of goats, cattle and sheep, and extraction of forest products; migration to cities of Mexico and the U. S. is also important in their economy [9].

### Production, incomes, and cultural aspects in homegardens

We studied a total of 30 homegardens, ten in each of the three first environmental zones referred to above, and considered and compared our information with that reported by Blanckaert *et al*. [20] for homegardens of the TSFZ. Homegardens studied were randomly sampled through a list of households in a village, assigning a number to them and then generating random numbers in a calculator. Semi-structured interviews [42] were conducted to the owners of each homegarden sampled, including questions on uses, names, management types, production and role of plant species of the system in the household’s subsistence. Native language is Náhuatl, after asking permit to carry out the study in communitarian meetings we interviewed 28 persons in Spanish since they were bilingual (Náhuatl and Spanish) and two persons only Náhuatl speakers were interviewed with the help of a local translator. Voucher specimens and photographs of each species recorded were prepared and information documented following the collecting format of the ethnobotanical data bank of Mexico, Banco de Datos Etnobotánicos de Plantas de México (BADEPLAN), of the Botanical Garden at UNAM.

We calculated the total amount of products obtained per homegarden through surveys and interviews about production per individual plant of a species, transforming the different local units of measurements (e.g. “caja”, “manojo”, “pieza”, “docena”, “bolsa”) in kg, and then by using data of vegetation sampling we estimated the total production in each homegarden. Proportions of products consumed directly by households and commercialized were estimated qualitatively by using as visual stimulus an image of a pie divided into five parts. We estimated incomes from the products of homegardens by investigating their prices through a survey carried out between August and October 2012, transforming their prices in Mexican pesos to U.S. dollars according to the exchange rate in that period. All the information referred to was stored in a database, and all quantitative analyses were conducted through the programme Past.

### Ecological parameters evaluated

We measured the area of each homegarden, constructed maps indicating the disposition of plant areas and other components of the systems, recording the number of individuals of each plant species within the whole homegardens. With this information we calculated the species richness, diversity and dominance per homegarden. Richness was determined as the total number of species per homegarden and then averaged this figure per environmental zone. Abundance was calculated as the total number of individual plants of each species per homegarden. Frequency was estimated as the number of individuals of a plant species with respect the total number of individual plants composing a homegarden. With these parameters we calculated the ecological importance index of each species per homegarden. Diversity was estimated by the Shannon-Wiener index*.* Then we calculated the dominance as a measure of representativeness of each species through the Simpson index. Equity, the proportion of the observed diversity with respect the maximum diversity expected was calculated through the Pielou index: ***J = H’ / H’ max***, in which ***J*** is Equity; ***H’*** = diversity; ***H’***_***max***_ = maximum diversity. ***H’max*** was calculated as the ***ln (S) S*** being the number of species in a sample. Similarity among the sampled units was estimated through the Jaccard index.

We sampled seven sites of natural vegetation (500 m^2^ each site); three in the CFZ, two in the TRFZ, and two in the TDFZ in order to compare their composition in perennial plant species with that of the homegardens studied. For conducting a similar comparison in the homegardens of the village of San Rafael we considered the information of two additional sites sampled by Vivar [41] in the Thorn-Scrub Forest locally called ‘jiotillal’ (dominated by the ‘jiotilla’ *Escontria chiotilla*) in the natural area around this village. With this information we determined the proportion of local diversity maintained within homegardens.

## Results

### Floristic composition and ecological parameters of homegardens

#### Species richness and diversity

**Figure 2:**
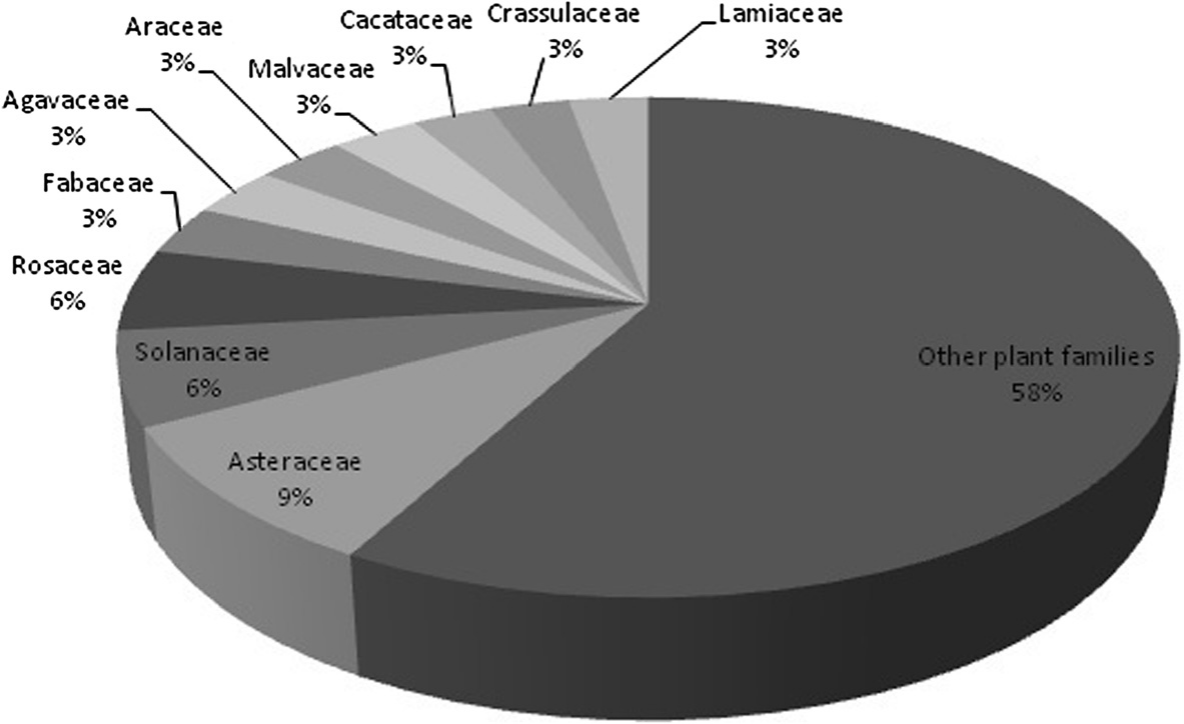
Plant families with the highest species richness recorded in the homegardens studied.

Area of homegardens in Coyomeapan and Ahuatla averaged 805 m^2^ ± D.S 550.98; in Aticpac 350 m^2^ ± D.S 168.33, whereas in Chimalhuaca and Yohuajca 300 m^2^ ± D.S 156.35. All parcels sampled are private property of the people interviewed. Most homegardens are in flatlands but in Coyomeapan and Ahuatla are on slight slope terrain. In the whole area sampled we recorded a total of 281 plant species belonging to 91 plant families, the richest one being Asteraceae (26 species), Solanaceae (17 species), Rosaceae (15 species) and Fabaceae (9 species) (Figure 2).

**Figure 3:**
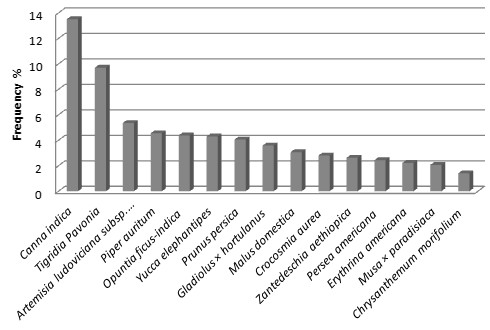
Frequencies of plant species recorded in the homegardens of Cloud Forest Zone (CFZ) at the communities of Coyomeapan and Ahuatla.

**Figure 4:**
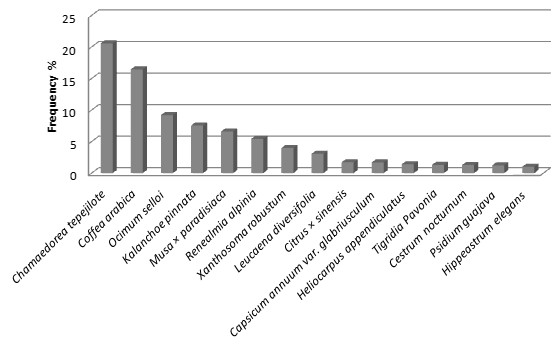
Frequencies of plant species recorded in the homegardens of Tropical Rainforest Zone (TRFZ) at the community of Aticpac.

**Figure 5:**
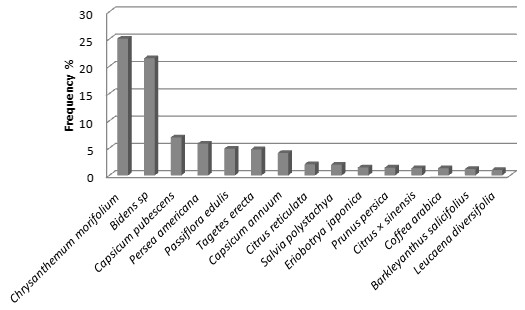
Frequencies of plant species recorded in the homegardens of Tropical Dry Forest Zone (TDFZ) at the communities of Chimalhuaca and Yohuajca.

**Figure 6:**
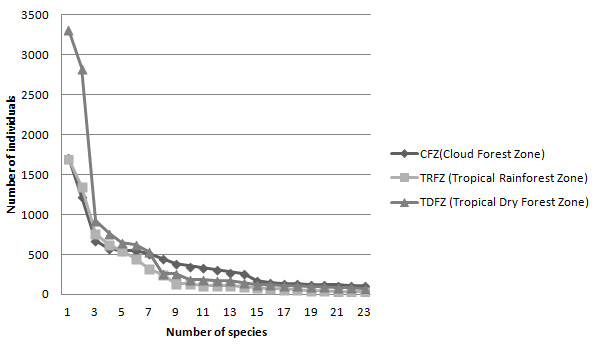
**Curve of species accumulated in vegetation sampling of homegardens, from higher to lower number of species at the communities of Coyomeapan, Ahuatla, Aticpac, Chimalhuaca y Yojhuaca.** Coyomeapan, Puebla. CFZ = Cloud Forest Zone, TRFZ = Tropical Rainforest Zone, TDFZ = Tropical Dry Forest Zone.

A total of 151 of the species recorded (34%) are native of the Tehuacán Valley, 20% are native in other areas of Mexico, and 130 (46%) are species introduced from other parts of the World. Herbs were the most numerous species (47%) in the homegardens studied, followed by trees (21%) and shrubs (20%). The remaining 12% of the species were other arborescent, vine, globose and cilindric cacti, and rosetophyllous plants. Figures 3, 4 and 5 shows species more frequently recorded in homegardens of the CFZ, TRFZ, and TDFZ. The highest species richness, diversity and equitability were recorded in homegardens of the CFZ, followed by those of the TRFZ, and then by those of the TDFZ (Table 1, Figure 6). This result contrasts with that found in natural vegetation; as it is shown in Table 2, the highest species richness and diversity were recorded in the Tropical Dry Forest (72 specie, ***H*** = 3.562), followed by the Tropical Rainforest (40 species, ***H*** = 2.74) and the Cloud Forest (24 species and ***H*** = 2.016). In the Thorn-Scrub Forest, Vivar (2004) recorded 69 plant species and estimated ***H*** = 1.28 (Table 2).

**Table 1:** Species richness, diversity (H’ = Shannon-Wiener index), dominance and equitability in homegardens of the studied zones

**Environmental zone**	**Home garden**	**Area (m**^**2**^**)**	**Number of plant species**	**Number of individual plants**	**Diversity (H’)**	**Dominance**	**Equitability**
**CFZ**	1	550	68	777	3.071	0.918	0.727
**CFZ**	2	1400	92	2687	3.041	0.918	0.672
**CFZ**	3	600	94	1116	3.092	0.884	0.68
**CFZ**	4	500	73	1384	2.675	0.833	0.623
**CFZ**	5	1000	89	1364	3.043	0.89	0.678
**CFZ**	6	2000	57	1184	2.81	0.906	0.695
**CFZ**	7	900	45	680	2.64	0.874	0.693
**CFZ**	8	100	66	2058	2.8	0.862	0.668
**CFZ**	9	400	46	801	3.225	0.944	0.842
**CFZ**	10	600	35	591	2.703	0.898	0.76
**χ**		**805**	**66.5**	**1264**	**2.91**	**0.89**	**0.70**
**σ**		**550.98**	**20.88**	**662.79**	**0.21**	**0.03**	**0.06**
**TRFZ**	11	400	91	1290	3.153	0.91	0.698
**TRFZ**	12	150	34	245	2.708	0.892	0.768
**TRFZ**	13	600	32	644	2.035	0.79	0.587
**TRFZ**	14	150	11	53	1.878	0.774	0.783
**TRFZ**	15	200	31	236	2.627	0.884	0.765
**TRFZ**	16	300	69	969	2.686	0.844	0.634
**TRFZ**	17	400	58	1587	2.126	0.783	0.523
**TRFZ**	18	250	47	736	2.308	0.817	0.599
**TRFZ**	19	600	39	1062	2.129	0.802	0.581
**TRFZ**	20	450	49	1420	2.195	0.795	0.564
**χ**		**350**	**46.10**	**824.20**	**2.38**	**0.83**	**0.65**
**σ**		**168.33**	**22.48**	**532.06**	**0.39**	**0.05**	**0.10**
**TDFZ**	21	500	43	574	2.801	0.898	0.744
**TDFZ**	22	600	37	444	2.578	0.857	0.713
**TDFZ**	23	250	14	47	2.427	0.895	0.919
**TDFZ**	24	200	34	2452	2.217	0.846	0.628
**TDFZ**	25	100	37	281	2.508	0.853	0.694
**TDFZ**	26	250	29	254	2.807	0.914	0.833
**TDFZ**	27	150	45	175	3.208	0.939	0.842
**TDFZ**	28	250	63	3933	1.966	0.768	0.474
**TDFZ**	29	400	19	1508	1.029	0.421	0.349
**TDFZ**	30	300	46	3537	1.007	0.35	0.262
**χ**		**300**	**36.70**	**1320.50**	**2.25**	**0.77**	**0.65**
**σ**		**156.35**	**14.07**	**1473.13**	**0.73**	**0.21**	**0.22**

CFZ = Cloud Forest Zone; TRFZ = Tropical Rainforest Zone; TDFZ = Tropical Dry Forest Zone.

**Table 2:** Species richness and diversity (Shannon-Wiener index) in natural vegetation of the studied zones

**Cloud forest zone**	**H’=**	**2.016**	**Species richness:**	**24 spp.**	
Species	Abundance	Relative frequency	Relative density	Relative dominance	Ecological value
*Quercus laurina*	380	15.625	34.959	26.899	77.483
*Quercus candicans*	261	11.458	24.011	23.774	59.244
*Ternstroemia sp.*	73	10.417	6.716	7.658	24.790
*Vaccinium leucanthum*	96	5.208	8.832	8.781	22.821
*Styrax argenteus*	96	5.208	8.832	7.812	21.852
*Baccharis conferta*	60	7.292	5.520	6.296	19.107
*Pinus sp.*	40	6.250	3.680	4.404	14.334
*Clethra sp.*	29	6.250	2.668	3.510	12.428
*Morella cerifera*	14	5.208	1.288	1.551	8.047
*Eupatorium sp.*	17	4.167	1.564	1.727	7.458
*Arbutus xalapensis*	11	4.167	1.012	1.320	6.499
*Quercus crassifolia*	6	4.167	0.552	0.713	5.431
*Rubus sp.*	2	2.083	0.184	0.231	2.498
*Litsea glaucescens*	2	2.083	0.184	0.225	2.492
**Tropical rain forest zone**		H’ = 2.744	Species richness:	40 spp.	
Species	Abundance	Relative frequency	Relative density	Relative dominance	Ecological value
*Chamaedorea tepejilote*	80	6.757	30.769	15.621	53.147
*Aphelandra scabra*	40	5.405	15.385	6.002	26.792
*Piper sp.*	19	6.757	7.308	6.346	20.411
*Guarea glabra*	11	5.405	4.231	8.450	18.086
*Parmentiera aculeata*	4	2.703	1.538	10.575	14.816
*Aphananthe monoica*	5	4.054	1.923	7.729	13.706
*Miconia aff. Argentea*	12	4.054	4.615	1.657	10.327
*Comarostaphylis sp.*	9	4.054	3.462	0.507	8.022
*Albizia sp.*	5	4.054	1.923	1.968	7.945
*Oreopanax xalapensis*	2	1.351	0.769	5.105	7.226
*Ruprechtia sp.*	1	1.351	0.385	5.016	6.752
*Maclura tinctoria*	2	1.351	0.769	4.074	6.194
*Heliocarpus appendiculatus*	3	4.054	1.154	0.333	5.541
*Coccoloba grandifolia*	2	2.703	0.769	1.336	4.808
*Parathesis sp.*	2	1.351	0.769	1.663	3.784
*Inga vera*	1	1.351	0.385	1.254	2.990
*Sapindus saponaria*	1	1.351	0.385	0.831	2.567
*Eugenia capulí*	1	1.351	0.385	0.495	2.231
*Dendropanax arboreus*	1	1.351	0.385	0.066	1.802
*Trema micrantha*	1	1.351	0.385	0.041	1.777
**Tropical dry forest zone**	H’=	3.562	Species richness:	72 spp.	
Species	Abundance	Relative frequency	Relative density	Relative dominance	Ecological value
*Lippia graveolens*	256	1.592	14.144	22.074	37.809
*Jatropha dioica*	94	2.387	5.193	9.261	16.842
*Dasylirion serratifolium*	26	2.122	1.436	11.479	15.037
*Dalea bicolor*	104	2.122	5.746	5.593	13.461
*Neopringlea sp*	74	2.122	4.088	6.014	12.224
*Gymnosperma glutinosa*	118	2.387	6.519	0.662	9.569
*Lippia sp.*	58	2.653	3.204	2.340	8.197
*Dodonaea viscosa*	70	2.653	3.867	0.988	7.508
*Calia secundiflora*	16	1.061	0.884	5.140	7.085
*Turnera diffusa*	76	1.326	4.199	1.186	6.711
*Calea sp.*	30	1.592	1.657	2.949	6.198
*Zaluzania sp.*	32	2.653	1.768	1.733	6.153
*Lysiloma acapulcensis*	46	2.653	2.541	0.898	6.092
*Eysenhardtia polystachya*	2	0.531	0.110	5.065	5.706
*Senna sp.*	46	2.122	2.541	0.953	5.616
*Galphimia glauca*	24	2.653	1.326	1.249	5.227
*Tecoma stans*	40	1.857	2.210	0.550	4.616
*Pilosocereus chrysacanthus*	10	2.122	0.552	1.560	4.235
*Opuntia pilífera*	12	2.122	0.663	1.347	4.132
*Ipomoea arborescens*	24	1.592	1.326	0.833	3.751
*Brickellia sp.*	10	2.122	0.552	1.004	3.679
*Mimosa sp.*	10	1.061	0.552	2.023	3.637
*Lantana cámara*	20	1.592	1.105	0.877	3.573
*Beaucarnea gracilis*	16	1.592	0.884	0.987	3.462
*Wimmeria sp.*	14	1.326	0.773	1.356	3.456
*Mammillaria sp.*	24	1.592	1.326	0.321	3.238
*Pittocaulon praecox*	10	2.122	0.552	0.547	3.221
*Indigofera sp.*	10	1.592	0.552	0.891	3.035
*Plumeria rubra*	8	1.592	0.442	0.659	2.692
*Senna fruticosa*	10	1.061	0.552	0.897	2.510
*Agave potatorum*	12	1.326	0.663	0.269	2.258
*Wimmeria microphylla*	20	0.531	1.105	0.451	2.086
*Pseudosmodingium sp.*	4	1.061	0.221	0.797	2.079
*Indigofera cuernavacana*	4	1.061	0.221	0.789	2.071
**Thorn-scrub forest zone**	H’=	1.28	Species richness:	69 spp.	
Species	Abundance	Relative frequency	Relative density	Relative dominance	Ecological value
*Mammillaria carnea*	127	14.85	8.44	0.42	23.71
*Gomphrena decumbens*	71	8.26	4.70	0.85	13.82
*Panicum* sp.	66	7.68	4.37	0.35	12.40
*Opuntia pilifera*	38	4.42	2.51	5.90	12.82
*Loeselia glandulosa*	34	3.99	2.27	0.05	6.30
*Dalea carthagenensis*	32	1.99	1.13	0.59	3.72
*Chamaesyce cumbrae*	32	3.69	2.10	0.16	5.95
*Euphorbia heterophylla*	28	3.28	1.87	0.08	5.22
*Zinnia peruviana*	24	2.81	1.60	0.29	4.70
*Pectis haenkeana*	22	1.93	1.10	0.46	3.50
*Mimosa luisana*	21	2.46	1.40	20.20	24.06
*Stenocereus stellatus*	21	2.42	1.38	1.25	5.05
*Coryphantha pycnacantha*	19	2.23	1.27	0.04	3.54
*Viguiera dentata*	17	0.82	0.47	2.27	3.56
*Dalea* sp.	17	3.75	2.13	1.58	7.47
*Escontria chiotilla*	17	1.99	1.13	20.81	23.94
*Phaseolus sp.*	17	2.58	1.47	0.11	4.16
*Opuntia puberula*	16	1.84	1.04	0.23	3.11
*Carminatia alvarezii*	14	1.64	0.93	0.06	2.64
*Lippia graveolens*	14	1.64	0.93	2.17	4.74
*Physalis philadelphica*	14	1.58	0.90	0.56	3.04
*Boerhavia erecta*	13	1.52	0.87	0.34	2.73
*Mimosa polyantha*	11	1.29	0.73	8.38	10.41
*Sanvitalia fruticosa*	10	1.17	0.67	0.06	1.90
*Croton* sp.	9	1.06	0.60	2.12	3.78
*Cordia curassavica*	9	1.02	0.58	2.52	4.11
*Celtis pallida*	8	0.94	0.53	0.60	2.07
*Commelina erecta*	8	0.94	0.53	0.04	1.52

CFZ = Cloud Forest Zone; TRFZ = Tropical Rainforest Zone; TDFZ = Tropical Dry Forest Zone; TSFZ = Thorn-Scrub Forest Zone (based on Vivar [41]). Only the most important species are reported.

#### Similarity of plant composition

Homegardens from the CFZ shared 108 species with those of the TRFZ (38.4% of the total number of species recorded in both areas), and 96 (34.1%) species with homegardens from the TDFZ. Homegardens from the TRFZ shared 86 (30.6%) species with those from the TDFZ. A total of 73 species (25.9% of all recorded in the whole sample) were shared among homegardens of the three zones directly studied.

### Benefits provided by homegardens

**Figure 7:**
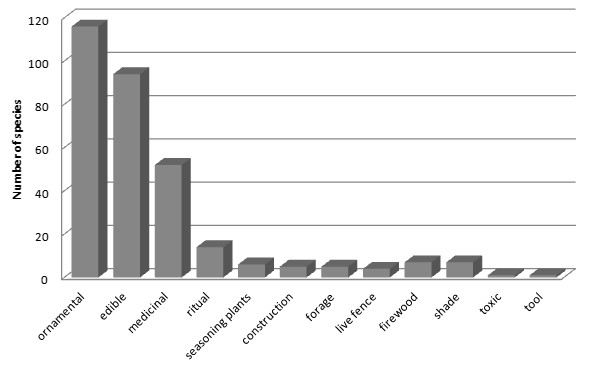
Uses of the plant species recorded in the homegardens of all the communities studied in the municipality of Coyomeapan.

According to perception of local people, homegardens provide the following main benefits: (1) complementing food, (2) closer availability of medicines, (3) obtaining monetary incomes, (4) complementing fuel wood and coffee, (5) obtaining shade, (6) soil retention, and (7) pleasure of being surrounded by ornamental beautiful plants. A total of 249 species were reported as having one use type, 30 species having two use types, and 2 species with three use types. The most numerous species were ornamental, followed by edible, medicinal, ritual, condiment, material for construction, living fences, fuelwood, shade, and tools (Figure 7).

A total of 89 native species were recorded in homegardens of the CFZ, most of them ornamental plants (39 species), followed by edible plants (32 species), medicinal plants (14 species) and other uses (14 species). In homegardens of the TRFZ we recorded 86 native species, most of them edible plants (32 spp.), followed by ornamental plants (28 spp.), medicinal plants (18 spp.), and other uses (16 spp.). In homegardens of the TDFZ we recorded 69 native species, most of them edible plants (28 spp.), followed by ornamental plants (18 spp.), medicinal plants (13 spp.), and other uses (16 spp., Table 3).

**Table 3:** Total number and percentage of cultivated, enhanced and tolerated native plant species with different use types recorded in all the homegardens studied

**Management type**	**Use**	**Number and percentage of species**
Cultivated	TOTAL	71 spp. (100.0%)
	Ornamental	35 spp. (49.2%)
	Edible	30 spp. (42.2%)
	Medicinal	7 spp. (9.8%)
	Others	4 spp. (5.6%)
Enhanced	TOTAL	23 spp. (100.0%)
	Ornamental	7 spp. (28.0%)
	Edible	11 spp. (44.0%)
	Medicinal	5 spp. (20.0%)
	Others	3 (12.0%)
Tolerated	TOTAL	57 spp. (100.0%)
	Ornamental	10 spp. (17.5%)
	Edible	8 spp. (14.0%)
	Medicinal	21 spp. (36.8%)
	Others	24 spp. (42.1%)

People surveyed said that most of the products from homegardens (67.1%) are destined to commercialization and the remaining part (32.9%) for direct consumption by household. A total of 43 species (15% of the total recorded) generate monetary incomes, but most species (238, 85% of the total recorded) are not interchanged. Main species from homegardens commercialized are indicated in Tables 4 and 5.

**Table 4:** Number of native plant species used with different purposes in the different environmental zones studied

**Zone**	**Use**	**Number of native plant species**	**% of native plant species recorded**
**Cloud forest**	Ornamental	39	43.8
	Edible	32	35.9
	Medicinal	14	15.7
	Others	14	15.7
	TOTAL	89	100
**Tropical rainforest**	Ornamental	28	32.6
	Edible	32	37.2
	Medicinal	18	20.9
	Others	16	18.6
	TOTAL	86	100.0
**Tropical dry forest**	Ornamental	18	26.1
	Edible	28	40.6
	Medicinal	13	18.8
	Othrs	16	23.2
	TOTAL	69	100.0

**Table 5:** Prices in U.S. dollars (rate change in August-October 2012) per commercialization unit and kg of useful plant products of species recorded in the homegardens studied which are traded in the markets of Coyomeapan

**Plant family**	**Species**	**Common name**	**Commercialization unit**	**kg/unit**	**Average price per unit**	**$/kg**
**ANNONACEAE**	*Annona cherimola*	Chirimoya	Caja	10	3.08	0.31
**ARECACEAE**	*Chamaedorea tepejilote*	Tepejilote	Manojo	0.5	0.38	0.76
**ASTERACEAE**	*Chrysanthemum morifolium*	Flor campechana	Docena	0.3	0.76	2.56
**CACTACEAE**	*Opuntia ficus-indica*	Nopal	Penca	0.6	0.38	0.64
**CARYOPHYLLACEAE**	*Dianthus caryophyllus*	Clavel	Docena	0.3	1.15	3.8
**CUCURBITACEAE**	*Sechium edule*	Chayote	Pieza	0.2	0.04	0.19
**LAURACEAE**	*Persea americana*	Aguacate	Caja	10	3.46	0.35
**MUSACEAE**	*Musa × paradisiaca*	Plátano	Kg	1	0.39	0.39
**MYRTACEAE**	*Psidium guajava*	Guayaba	Bolsa	0.4	0.39	0.96
**PASSIFLORACEAE**	*Passiflora edulis*	Granadilla	Caja	10	6.15	0.62
**ROSACEAE**	*Malus domestica*	Manzana	Caja	10	3.65	0.37
**ROSACEAE**	*Prunus persica*	Durazno	Caja	10	4.62	0.46
**ROSACEAE**	*Eriobotrya japonica*	Níspero	Caja	10	3.85	0.39
**ROSACEAE**	*Prunus serotina*	Capulín	Bolsa	0.5	0.39	0.77
**ROSACEAE**	*Prunus domestica*	Ciruela	Caja	10	2.69	0.27
**RUBIACEAE**	*Coffea arabica*	Café tostado y molido	Kg	1	6.92	6.92
**RUBIACEAE**	*Coffea arabica*	Café verde	Kg	1	3.85	3.85
**RUTACEAE**	*Citrus reticulata*	Madarina	Bolsa	1	0.39	0.39
**RUTACEAE**	*Citrus × sinensis*	Licor de naranja	Lt.	1	3.08	3.08
**RUTACEAE**	*Citrus × sinensis*	Naranja	Kg	1.25	0.39	0.31
**RUTACEAE**	*Citrus x aurantifolia*	Lima	Bolsa	1	0.39	0.39
**SOLANACEAE**	*Capsicum pubescens*	Chile canario	Caja	10	3.46	0.35
**ZINGIBERACEAE**	*Renealmia alpinia*	Belígmoli	Docena	0.1	0.39	3.85

Other species such as apple (*Malus domestica*), prunes (*Prunus domestica*), peaches, canario chili (*Capsicum pubescens*), granadilla or passion fruit (*Passiflora edulis*) and custard apple (*Annona cherimola*) are also commercialized in markets, but these are mainly traded to foreign hoarders in the place of Coyomeapan. Hoarders pay low and unfair prices to local people for their products to commercialize them at higher prices in markets of the cities of Tehuacán, Ajalpan, and Zoquitlán in the state of Puebla, Zongolica, in the state of Veracruz and Teotitlán, in the state of Oaxaca. Barter is also a common practice exchanging a variety of products for maize and coffee.

### Plant management

#### Management types

A total of 176 species recorded in the homegardens studied are cultivated, 71 species are native tolerated or let standing and 34 are enhanced or promoted. A total of 151 of the managed plant species are native (71 cultivated, 57 tolerated, and 23 enhanced, Tables 3 and 4). It can be noticed that the sum of the species mentioned is higher than the total recorded, because several species have more than one management type.

#### Spatial arrangement

A general pattern of spatial arrangement of plant species in homegardens were identified. Herbaceous, shrubby and vine plants providing benefits as ornamental, medicinal, and condiment are placed close to the houses, because according to local people these species generally require irrigation, they make beautiful their houses and provide medicines promptly when needed. Some tall trees are also placed close to the house, mainly *Alnus acuminata* (30% of all homegardens sampled), *Quercus* spp. (23%), *Cupressus* spp. (20%), *Fraxinus uhdei* (20), *Salix taxifolia* (13%), and *Platanus mexicana* (7%); according to people, these are trees destined to provide shade to the houses and to protect them against strong wind. Fruit producing trees and other large tree species are placed distanced from houses because, according to people, these species have extended roots that may affect the houses’ floor, and because their eventual falling down may destroy the house. In Coyomeapan and Ahuatla (CFZ) homegardens are delimited by living fences more commonly formed with *Erythrina americana* (50% of all homegardens of the CFZ), *Yucca elephantipes* (40%), *Brugmansia candida* (40%), *Quercus laurina* (23%), *Fraxinus uhdei* (20%), *Arundo donax* (20%), *Cupressus* spp. (20%), and *Jasminum fruticans* (17%). In Aticpac (TRFZ) people construct fences with stones and *Hibiscus rosa-sinensis* (50% of all homegardens of this village), whereas in Chimalhuaca and Yohuajca (TDFZ) there are no living fences.

#### Maintenance of homegardens

Women are the main managers of homegardens, practicing sowing, planting, maintenance and harvest of most products; also they are the main responsible of trading their products. Men participate in activities such as tree pruning, weeding, fertilization and actions against pests and harvesting of some products, mainly those of tall trees. Widows use to pay a salary (nearly $6.50 U.S. dollars per day) to men for doing these activities.

People from Coyomeapan, Ahuatla, Chimalhuaca and Yohuajca (CFZ and TDFZ) use to regularly add as fertilizer ground collected in the forest (‘tierra de monte’), ash from home fire, dung of hens, sheep and goats. In Aticpac (TRFZ) people leave the fallen leaves of plants to recycling into the soil and occasionally add forest ground to some particular plants, mainly fruit trees.

The general opinion of local people is that pests are not a real problem; however, people from Coyomeapan and Ahuatla (CFZ), add lime to fruit trees trunks in order to prevent ants and aphid attack; this method also allows controlling lichen growing since people consider that lichens “robe life to trees” affecting fruit production. Branches of big trees such as oaks, alder and ash trees are pruned when the hemi-parasite plant called “tempala” (*Phoradendron* sp.) infests them. In Aticpac (TRFZ) people use to remove by hand worms infesting plants in homegardens; ants and “cenicilla” (*Oidium* sp.) are prevented by putting lime on tree trunks. In Chimalhuaca and Yohuajca (TDFZ) people make use of agrochemical products to prevent pest attack since they cultivate ornamental plants close to the homegardens and they consider that pests of those crops will extend rapidly to homegardens.

Pruning is also practiced to control tree growing in order to make harvesting easy. Herbaceous and shrubby plants close to the houses are irrigated every three days during the dry season. Fruit trees are only occasionally irrigated.

## Discussion

Our study confirmed that the homegardens studied harbour a high biological diversity represented by a total of 281 plant species. Although lower than other traditional agroforestry systems of the region (see for instance [7,29]), 34% of the plant species maintained in homegardens are native to the region and 16% are native to local vegetation of the territories analysed. Such diversity reflects in first term the cultural interest of local people for maintaining multiple options for complementing their subsistence patterns, including food and other needs security. The local human cultures studied, as the Mesoamerican peoples in general, have solved their subsistence needs by the multiple forms of using natural resources and ecosystems [43]. But the high diversity recorded in this study also reflects the high biodiversity of natural ecosystems of the Tehuacán Valley, one of the highest biodiverse regions of Mexico [34]. Several authors (see for instance [44]), have discussed that homegardens commonly resemble the structure of natural ecosystems. The diversity in composition, structure and functions are human constructions and are apparently inspired in the surrounding ecosystems.

We should highlight that along with the local plant species diversity maintained in homegardens there is also an associated diversity [5] involving other groups of organisms (e.g. birds, insects, mammals) which we have not evaluated yet but that find in homegardens favourable habitats for reproducing their lives. General diversity (not only that of native plant species) is in theory highly relevant for the system resilience, and local and regional policies for biodiversity conservation have in homegardens targets for enhancing their richness and composition with particularly endangered species or those that could favour local associated diversity.

Species richness, diversity and equitability were higher in homegardens of the CFZ, followed by those of the TRFZ and then those of the TDFZ. Contrarily to our expectations, the highest diversity was recorded in homegardens where the neighbouring forests had the least diversity and vice versa. This pattern suggests that local people manage in homegardens mainly plant species that are not available in the wilderness close to their towns; at least for some species management appears to aim at compensating scarcity of naturally available plant resources. Based on sampling of natural vegetation we found that homegardens share with the local forests a total of 25 perennial plant species (Table 6), but nearly 100 species recorded are native to the region. In other words, although the capacity of homegardens to conserving local diversity is relatively low, their capacity for conserving regional biodiversity is high.

**Table 6:** Plant species shared among homegardens and natural vegetation in the studied zones at Coyomepan, Puebla

	**CFZ**	**TRFZ**	**TDFZ**	**TSFZ**
	*Chamaedora elegans*	*Cercocarpus macrophyllus*	*Acacia farnesiana*	*Acacia cochliacantha*
	*Platanus mexicana*	*Chamaedora elegans*	*Salix taxifolia*	*Acalypha* sp.
	*Plumeria rubra*	*Hamelia patens*		*Celtis pallida*
	*Rubus eriocarpus*	*Rubus eriocarpus*		*Cercidium praecox*
	*Salix taxifolia*	*Talauma mexicana*		*Commelina erecta*
		*Siparuna andina*		*Escontria chiotilla*
				*Ferocactus latispinus*
				*Lippia graveolens*
				*Physalis philadelphica*
				*Sedum* sp.
				*Stenocereus pruinosus*
				*Ziziphus amole*
Total: 25	5	6	2	12
*Percentage	18.50%	22.22%	7.40%	^+^5.05%

* Percentage calculated based on the total number of species of trees and shrubs recorded in vegetation sampling of homegardens and natural vegetation in localities of Coyomeapan.^+^ Percentage calculated based on the total number of species of trees, shrubs and herbs recorded in homegarden’s vegetation sampling by Blanckaert *et al*. 2004 and wild jiotillal ’s vegetation sampling by Vivar (2004) in San Rafael Coxcatlán. CFZ = Cloud Forest Zone, TRFZ = Tropical Rainforest Zone, TDFZ = Tropical Dry Forest Zone; TSFZ = Thorn-Scrub Forest Zone.

Composition of homegardens seems to be related with the local environmental conditions of the villages studied; those from CFZ, TRFZ and TDF zones are different in composition among zones but similar within each zone. Composition of the homegardens studied in Coxcatlán by Blanckaert *et al*. [20] is in turn markedly different to that recorded in our study. For instance, Blanckaert *et al*. [20] found that cacti are among the main components of this system. Such composition is influenced by the high cultural value of cacti [17,23] but also because of their adaptations to the local semiarid environment in Coxcatlán. Homegardens of the CFZ are significantly larger than those of the other zones studied, and that this aspect may influence composition of the system; however, according to [26] all the homegardens studied are within the ‘small size’ category.

Social factors also influence homegardens’ composition. Although all the villages studied are neighbouring territories inhabited by Náhuatl people with similar culture, the economic purposes of homegardens vary among villages. Production of food (and monetary incomes derived from these products), medicines and ornamental are the most important purposes, and these factors guide criteria for making decisions about the composition of homegardens, but they are different among the villages studied as well as to those of Coxcatlán. For instance, in homegardens of the CFZ medicinal plants are scarcer than in the other zones. This could be associated to the decreasing importance of traditional medicine in communities of that zone which have public health centres and private clinics as well as transportation to urban centres. For the contrary, in those communities edible and ornamental plant species for commercialization are more important. In the communities of the other zones studied medical services are more deficient and traditional medicine more important, as well as composition of medicinal plants in homegardens. In communities of the TDFZ people cultivate ornamental plants for commercialization and this group of plants is therefore more important than others. Similarly, Blanckaert *et al*. [20] found that in Coxcatlán ornamental plant species of the families Araceae and Liliaceae are particularly important. In fact, ornamental purpose is relatively more important in Coxcatlán than in all villages of Coyomeapan (Table 7). These patterns show that homegardens are systems influenced by ecological conditions and restrictions, but also by cultural and economic aspects configuring the role of the system in local people’s subsistence.

**Table 7:** **Main plant families and species richness recorded in Coyomeapan (this study) and San Rafael, Coxcatlán (according to Blanckaert et al.**[20]**)**

**Municipality**	**Total number of plant species**	**Plant family**	**Number of plant species**
Coyomeapan	281	Asteraceae	26
		Solanaceae	17
		Rosaceae	15
		Fabaceae	9
		Agavaceae	8
Coxcatlán	233	Araceae	15
		Cactaceae	14
		Liliaceae	13
		Solanaceae	12
		Crassulaceae	10

The area comprised in this study is a relatively small portion of the great diversity of biocultural contexts of the Tehuacán Valley and it is far to be representative of the region. We reported information for only four of a total of 36 types of vegetation [45], and for one of eight indigenous ethnic groups of the region [16]. It is therefore possible to expect a high diversity of settings at the regional level yet to be studied. The method carried out allowed a relatively rapid diagnostic that could be implemented in short time for sampling the different biocultural conditions of the Tehuacán Valley, which would allow constructing strategies for regional biodiversity conservation and sustainable management. What is particularly relevant from our current study is the fact that the homegardens described are the expression of a current capacity for maintaining general diversity, and their important role for satisfying needs of local peoples.

Plant management involves important traditional ecological knowledge, practices, and technical experiences for designing any management plan. Most managed species were recorded in homegardens of CFZ, followed by those of TRFZ and TDFZ. Most species recorded are cultivated, followed by tolerated and enhanced plants. Most cultivated species are ornamental plants and edible species and only nearly 10% are medicinal plant; however most of the tolerated species are medicinal plants. Most studies on plant management have been centred in edible plants, but the results from this study suggest that studies on management and domestication should put more attention to ornamental and medicinal plants. Most enhanced or promoted plant species are edible and clearly this management type is directed to increase this benefit (Table 8). Although only 15% of all plant species recorded are commercialized, production in homegardens is continuous allowing monetary incomes throughout the year.

**Table 8:** **Percentage of native plant species that are managed and used in different forms in the municipalities of Coyomeapan (this study) and San Rafael Coxcatlán, Puebla (according to Blanckaert et al.**[20]**)**

**Municipality**	**Management type**	**%**	**Use**	**%**
Coyomeapan	Cultivated	63	Ornamental	49.2
			Edible	42.2
			Medicinal	9.8
	Protected	12	Ornamental	28
			Edible	44
			Medicinal	20
	Tolerated	25	Ornamental	17.5
			Edible	14
			Medicinal	36.8
Coxcatlán	Cultivated	68	Ornamental	70
			Edible	29.5
			Medicinal	6.5
	Protected	10	Ornamental	47.6
			Edible	36.5
			Medicinal	15.9
	Tolerated	22	Ornamental	55.2
			Edible	31
			Medicinal	17.2

According with Altieri [27], traditional management systems may be adapted for increasing productivity and sustainability. For such purpose it is particularly relevant promoting conservation of diversity in agroecosystems as much as possible. Such purpose may increase the potential contribution of these systems to biodiversity conservation, food sufficiency, and ecological functions that favour higher resilience capacity and lower vulnerability to natural or socio-economic and cultural contingencies. Based on the information reported, it is possible to affirm that local homegardens are important reservoirs of biodiversity and that although local native biodiversity maintained within them is relatively lower than in other agroforestry systems, they may significantly contribute to its conservation at regional level. Such capacity should also be seen at landscape level. Considering that these systems may harbour endemic threatened species, they should be included in the strategies of biodiversity conservation and human wellbeing at regional level of the important biosphere reserve Tehuacán-Cuicatlán.

## Conclusions

Homegardens studied in the municipality of Coyomeapan are reservoirs of high plant species diversity, nearly 34% of it being native to the Tehuacán Valle and nearly 16% to the local vegetation. The highest diversity was recorded in homegardens where the neighbouring forests had the least diversity, which suggests that management of homegardens aims at compensating scarcity of naturally available plant resources. Differently to other agroforestry systems of the area, cultivated species were markedly more abundant than plants under other management forms. Homegardens’ composition is influenced by ecological conditions and social factors according the role of the system in local people’s subsistence.

The information documented may support local programs for agroecological practices linked to dynamic conservation of biodiversity and culture. Homegardens may be important for local and regional strategies of protection of threatened species along with those of economic importance. Promoting interchange of local experiences about use and management techniques among rural communities, as well as diffusion of ecological and cultural information about the species managed could strongly support such a process. Academic institutions and NGOs might contribute with scientific and regional and national management experiences for making decisions at different scales.

## Competing interests

The authors declare that they have no competing interests.

## Authors' contributions

CL main author, involved in the study design, conducting of interview, field work, literature review and general data collection and systematization, wrote the first draft and concluded the final version this paper. AC main coordinator-supervisor of the research project; contributed with original data and the designing of all the researches providing the information for the current analysis; participated in fieldwork, systematization and analysis of data and reviewed several drafts of the manuscript. MV AIM and JB contributed to designing and following progress of the research and field work and data analyses. All authors read and approved the final manuscript.

## Authors' information

CL Undergraduate student at the Centro de Investigaciones en Ecosistemas (CIEco), AC, full time researchers at CIEco, UNAM. AIM associate professor at the Escuela Nacional de Estudios Superiores, UNAM, MV and JB postgraduate student at the Centro de Investigaciones en Ecosistemas (CIEco), UNAM.
